# Characterization and DNA Stable-Isotope Probing of Methanotrophic Bioaerosols

**DOI:** 10.1128/spectrum.03421-22

**Published:** 2022-11-21

**Authors:** Kevin P. Dillon, Valdis Krumins, Aishwarya Deshpande, Lee J. Kerkhof, Gediminas Mainelis, Donna E. Fennell

**Affiliations:** a Department of Environmental Sciences, Rutgers University, New Brunswick, New Jersey, USA; b Department of Biochemistry and Microbiology, Rutgers University, New Brunswick, New Jersey, USA; c Department of Marine and Coastal Sciences, Rutgers University, New Brunswick, New Jersey, USA; University of Massachusetts Amherst

**Keywords:** bacterial growth, aerobiology, methane oxidation, humidity, water availability

## Abstract

The growth and activity of bacteria have been extensively studied in nearly every environment on Earth, but there have been limited studies focusing on the air. Suspended bacteria (outside of water droplets) may stay in the atmosphere for time frames that could allow for growth on volatile compounds, including the potent greenhouse gas methane. We investigated the ability of aerosolized methanotrophic bacteria to grow on methane in the airborne state in rotating gas-phase bioreactors. The physical half-life of the aerial bacterium-sized particles was 3 days. To assess the potential for airborne growth, gas-phase bioreactors containing the aerosolized cultures were amended with 1,500 ppmv ^13^CH_4_ or ^12^CH_4_. Three of seven experiments demonstrated ^13^C incorporation into DNA, indicating growth in air. Bacteria associated with the genera *Methylocystis* and *Methylocaldum* were detected in ^13^C-DNA fractions, thus indicating that they were synthesizing new DNA, suggesting growth in air. We conclude that methanotrophs outside of water droplets in the air can potentially grow under certain conditions. Based on our data, humidity seems to be a major limitation to bacterial growth in air. Furthermore, low biomass levels can pose problems for detecting ^13^C-DNA synthesis in our experimental system.

**IMPORTANCE** Currently, the cellular activities of bacteria in the airborne state outside of water droplets have not been heavily studied. Evidence suggests that these airborne bacteria produce ribosomes and metabolize gaseous compounds. Despite having a potentially important impact on atmospheric chemistry, the ability of bacteria in the air to metabolize substrates such as methane is not well understood. Demonstrating that bacteria in the air can metabolize and grow on substrates will expand knowledge about the potential activities and functions of the atmospheric microbiome. This study provides evidence for DNA synthesis and, ultimately, growth of airborne methanotrophs.

## INTRODUCTION

Microbes are important mediators of elemental cycling in nearly all environments in which they are present. However, it is not known if microbes participate in elemental cycling in the alfresco atmosphere. There are 10^4^ to 10^5^ microbes/m^3^ of air (1) in the atmosphere that are derived from various sources and return to the Earth’s surface through wet or dry deposition (2). In the atmosphere, microbes can be suspended in water droplets (e.g., clouds), adhered to particles, or suspended planktonically in air. While mixed with water droplets, microbes in clouds have the potential to affect *in situ* cloud physics and chemistry (3) and cloud water can support microbial growth (4). In laboratory experiments, it has been demonstrated that microbes in airborne water droplets can metabolize glucose (5), synthesize new DNA (6), and divide (7, 8). However, the major part of the atmosphere does not contain water droplets, and the bacteria are usually either attached to particles or present as single cells suspended in air (9). It has been found that bacteria associated with larger particles in the air were less viable on average than single bacterial cells in the air (10).

Recent studies have indicated that microorganisms that are suspended in the air outside of water droplets have the potential to be active. Bacteria adhered to Saharan dust were thought to actively metabolize nitrogenous compounds (11). Furthermore, airborne bacteria in urban and indoor environments were determined to have active enzymes (12–14). Additionally, bacteria present in outdoor air have elevated RNA/DNA ratios, which indicates potential cellular activity (15–17). This activity could involve cellular maintenance, stress responses, substrate metabolism, or possibly growth. When supplied with volatile organic compounds (VOCs) and humid conditions, airborne bacteria are able to synthesize ribosomes (18), which is a prerequisite for growth. Various VOCs in the air are present at sufficient concentrations for microbial growth (19). Based upon this, the atmosphere could be an environment in which microorganisms metabolize VOCs and grow.

To study microbial activity and growth, various techniques are used that range from “omic” analyses (3, 20) and enzymatic assays (13, 21) to the incorporation of stable isotopes (e.g., ^13^C) (22, 23). A common application of the latter approach is DNA stable-isotope probing (DNA-SIP), which is used to study microbial growth on a specific substrate (24). The microbes need to produce new DNA and replicate, typically twice, to be detected by DNA-SIP. The doubling time of microbes in the natural environment has been estimated based on the combination of accumulated mutations and measured mutation rates to be between 1 and 25 h for certain model organisms (25). Microbes can be suspended in the atmosphere for days to weeks, depending on the characteristics of aerosolized cells and meteorological conditions (26), which allows enough time for theoretical microbial growth in the atmosphere. Single cells in the atmosphere have access to volatile compounds, making the question of their ability to grow on these compounds pertinent.

An important gas that has been increasing in concentration over the last few decades due to anthropogenic activities is methane, the concentration of which is over 1,800 ppb on average in the atmosphere (27, 28). However, there are methane hot spots in which the ambient local methane concentration can be increased by thousands of parts per billion, depending upon local emissions and meteorological conditions (29). The main sink for methane in the atmosphere is reactions with radicals (30). However, an important sink for this gas in terrestrial and aquatic systems is biological, through the activity of methane-oxidizing bacteria (methanotrophs) (31), which are common in soils, rice paddies, and the phyllosphere (32, 33). Among methanotrophs, the most well known are within the orders *Methylococcales* (*Gammaproteobacteria*) and *Rhizobiales* (*Alphaproteobacteria*), which are also referred to as type I and type II methanotrophs, respectively (34). Beyond phylogeny, these groups differ according to cellular structure and biochemistry (35). Methanotrophs can also be categorized depending upon the concentration of methane that they can oxidize. High-affinity methanotrophs can oxidize methane at low concentrations (e.g., atmospheric levels), while low-affinity methanotrophs oxidize methane at higher concentrations. High-affinity methanotrophs include the USCα clade of type II methanotrophs, such as Methylocapsa gorgona MG08, which can grow on atmospheric concentrations of methane (36). Some methanotrophs can oxidize atmospheric methane concentrations when first provided a higher concentration of methane (37). As a result, the concentration of methane in the environment dictates the types of methanotrophs that are able to oxidize it.

The presence of methanotrophs in these different environments has wide-ranging implications for the Earth’s methane budget (31). From wetland soils to tree bark, methanotrophs can be important removers of methane (33, 38). Methanotrophs can also be found in the atmosphere and have been demonstrated to be active under cloud-like conditions (39). As discussed, various heterotrophic bacteria can be active in the air, so it is possible that airborne methanotrophs oxidize and grow on methane in the air outside of water droplets (40). The goal of this study was to address this possibility. We sought to characterize bioaerosol particles produced from methanotrophic enrichment cultures and to determine by DNA-based SIP if methanotrophs synthesize ^13^C-DNA in the airborne state when provided with ^13^C-methane. Because DNA synthesis represents the final process before cellular division, our findings provide strong evidence that aerosolized methanotrophs can grow while suspended in the air.

## RESULTS

### Enrichment of methanotrophs.

**FIG 1:**
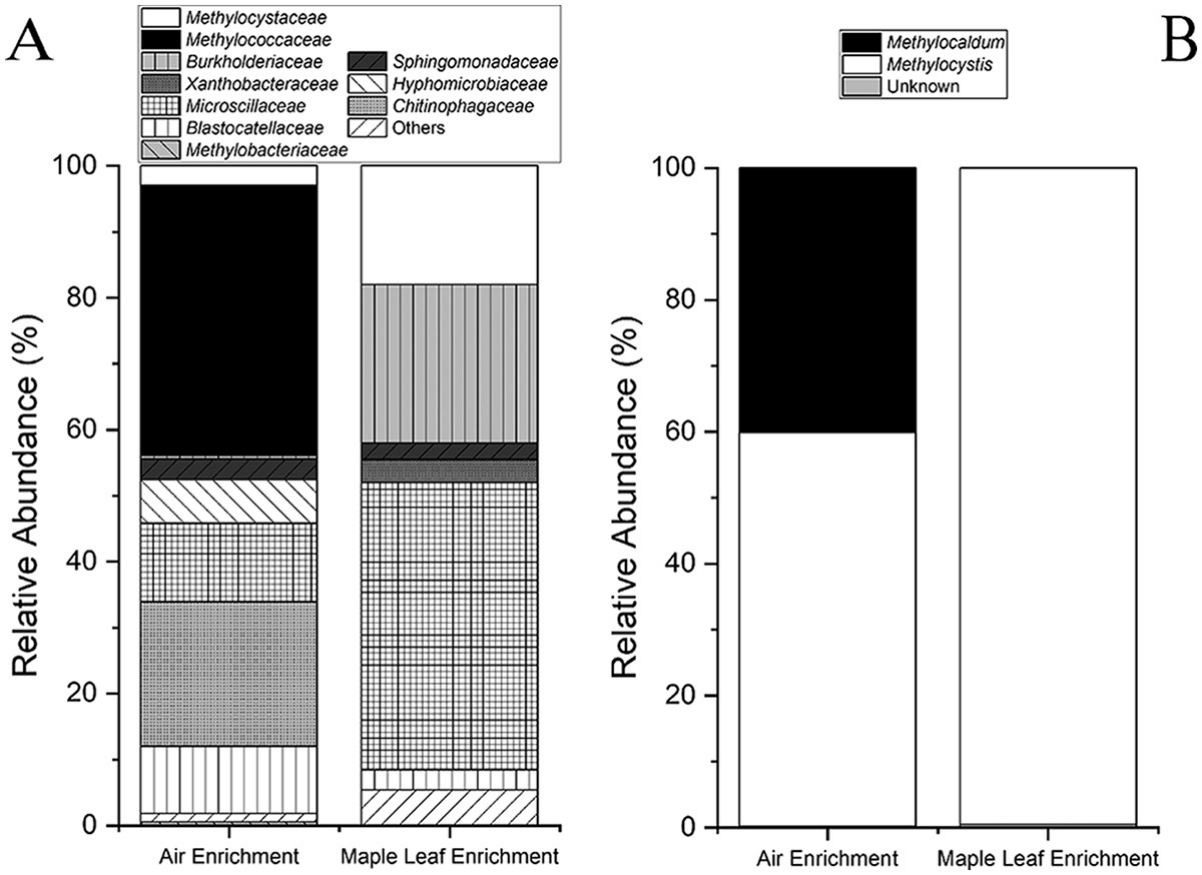
Bacterial compositions of the methanotrophic air enrichment and methanotrophic maple leaf enrichment cultures. (A) Relative abundances of the different ASVs at the family level based upon partial 16S rRNA gene sequences. (B) Compositions of the cultures at the genus level for methanotrophs present in the cultures based upon partial *pmoA* gene sequences.

Microbial growth was observed in both the air and maple leaf enrichment cultures on 4% (vol/vol) methane by measuring a decrease in methane concentrations and an increase in optical density at 600 nm (OD_600_) (see Fig. S1A and S1B in the supplemental material). Community analyses (Fig. 1) indicated that 41% of the 16S rRNA gene amplicon sequence variants (ASVs) in the air culture were related to the family *Methylococcaceae* (type I methanotroph). In the maple leaf enrichment culture, 18% of the 16S rRNA gene ASVs were related to the *Methylocystaceae* (type II methanotroph) (Fig. 1A). The remainder of the community was related to taxa not known to be methanotrophs. Among these other taxa, the methylotrophic *Methylobacteriaceae* were present at about 0.5% in the air culture only. Members of this family are not methanotrophic but are known to metabolize other compounds in the methane oxidation pathway (e.g., methanol). Various other heterotrophic bacteria were present in the enrichment cultures (Fig. 1A). Based upon *pmoA* gene sequences, the air enrichment culture contained *Methylocystis* and *Methylocaldum*, while the maple leaf enrichment culture contained only *Methylocystis* (Fig. 1B).

### Bioaerosol particle behavior.

**FIG 2:**
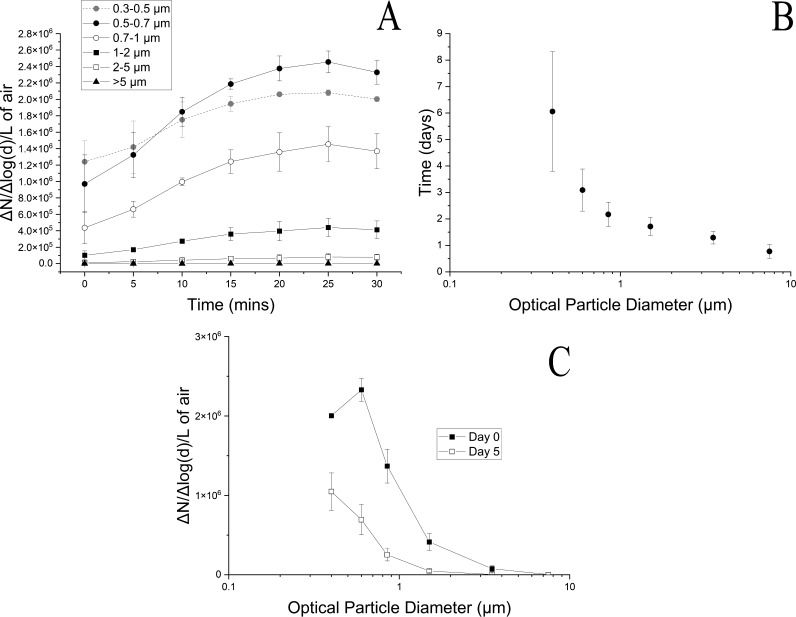
Bioaerosol characteristics. Two gas-phase bioreactors rotating at 1 rpm were filled with bioaerosols of a mixture of a methanotrophic air enrichment and *S. aerolata* NW12 for 30 min in 5-min increments. (A) Bioaerosols are characterized by the filling profile showing particle size distribution in the bioreactors; (B) physical retention (half-life) of particles in different particle size bins over a 5-day period; (C) average particle size distribution at days 0 and 5.

When the cultures were aerosolized into the bioreactors, the particles increased in concentration over time (Fig. 2A). Particle size distributions are presented as the number of particles normalized to the logarithmic difference between the upper and lower size bins. The particle size distribution reached a potential dynamic equilibrium at 25 min (Fig. 2A). The particle size distribution peaked in the 0.5- to 0.7-μm size bin, with a midpoint optical diameter of 0.6 μm. However, the particles in this size bin could be salts from the growth medium, as well as bacteria. We found that even if the cell suspension was washed three times with sterile water, thus removing salts, the particle size distribution value in the 0.5- to 0.7-μm size bin was the greatest (Fig. S2). This value was about half of that of the unwashed cell suspension (Fig. S2). Based upon this observation, bacteria were likely detected in this size bin for optical diameter.

Once particles were introduced into the bioreactors, the particle half-lives were assessed over a 5-day period (Fig. 2B). The bacterium-sized particles had a half-life (referring to physical retention of particles, not survival) of about 3 days at 3 rpm. The number of particles in the bioreactors decreased daily (Fig. 2C). Based on the size distributions, at day 0 there were 2.3 × 10^6^ ± 1.4 × 10^5^ Δ*N*/Δlog(*d*)/L particles and 6.9 × 10^5^ ± 1.9 × 10^5^ Δ*N*/Δlog(*d*)/L particles in the 0.5- to 0.7-μm size bin. In particle size distributions, N refers to the concentration of the particles in a specific size bin and *d* refers to the particle diameter for the same size bin. The particle concentrations for these size distributions are presented in Table S2. Bacteria were culturable on DSMZ 921, a nitrate mineral salts medium, after aerosolization and after 5 days of incubation (Fig. S3). Immediately after aerosolization, the ratio was 1.94 × 10^−2^ ± 6.37 × 10^−3^ CFU/particle initially, and it decreased to 3.12 × 10^−3^ ± 3.19 × 10^−4^ CFU/particle after 5 days, a significant decrease (*P* = 1.36 × 10^−3^) (Fig. S3).

### Liquid-phase DNA stable-isotope probing.

**FIG 3:**
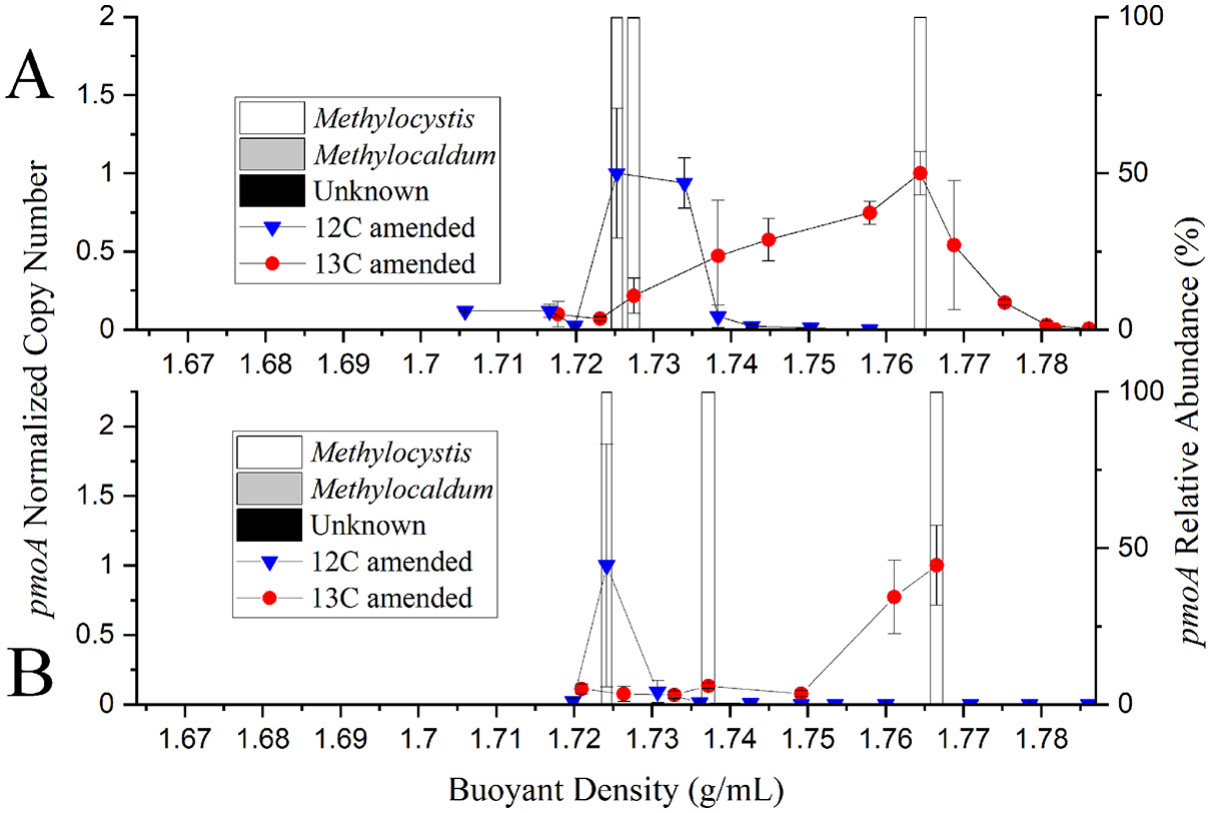
Liquid-phase growth of methanotrophs. (A and B) Duplicate sets of the methanotrophic air enrichment culture in liquid medium incubated with 4% [^12^C]methane or [^13^C]methane (vol/vol, gas headspace) over 6 days. The ^12^C- and ^13^C-labeled DNA was separated by ultracentrifugation and gradient fractionation. The presence of the DNA of methanotrophs across the density gradient was detected by qPCR of the *pmoA* gene (normalized to the highest copy number of the *pmoA* gene in a single sample). The *pmoA* amplicons of fractions at select densities were sequenced, and the relative abundances of different genera were identified as *Methylocystis*, *Methylocaldum*, or unknown based upon *pmoA* sequencing.

To benchmark subsequent experiments in the gas-phase bioreactors using aerosolized cells, we incubated duplicate sets of the air enrichment culture in liquid medium in the presence of [^13^C]methane or [^12^C]methane (Fig. 3). This served as a control to ensure that SIP analytical procedures were working under a best-case scenario of high biomass and replicating cells. Sequences of *pmoA* gene amplicons were classified into ASVs according to association with different genera of methanotrophs, and relative amounts were plotted versus the buoyant density of the fractions. *Methylocystis* was dominant in DNA recovered from cultures fed [^12^C]methane and [^13^C]methane (Fig. 3). Unknown *pmoA* sequences and *pmoA* sequences associated with *Methylocaldum* were detected in trace relative abundances. These PmoA sequences clustered with known *Methylocystis* and *Methylocaldum* species in phylogenetic analyses as well (Fig. S4). Based upon our data from the liquid-phase DNA-SIP, we detected ^13^C-labeled DNA and ^12^C-labeled DNA. Results of DNA-SIP indicate that methanotrophs were actively growing on methane while in the liquid phase.

### Gas-phase DNA stable-isotope probing.

Table 1 provides an overview of the experiments conducted for gas-phase DNA stable-isotope probing. The temperatures in the reactors after aerosolization (day 0) and upon sampling (day 5) ranged from 18 to 24°C. The total amount of DNA (inferred from concentrations) recovered at the end of airborne incubation varied across the experiments. Reactor 1 in experiment 7 had 34.2 ng DNA as the maximum DNA mass, while the DNA amount in experiment 3 was below the detection limit (estimated to be 0.2 ng) of the Qubit 2.0 fluorometer (Table 1). After the 5-day incubation period, there were 105 particles/L when the air or maple leaf enrichments were present in the bioreactors. The particles in the reactors were not all bacterial cells, as salts from the growth medium were also present (Fig. S2). The estimated methanotrophic cell numbers are based upon the cumulative total of *pmoA* genes detected by quantitative PCR (qPCR) for analyzed fractions. After the 5-day incubation period, the number of methanotrophic cells ranged from none detected to a maximum of 3.01 × 10^5^ cells in total for bioreactor 2 of experiment 5 (Table 1). Upon sampling (day 5), the relative humidity in the reactors ranged from 78 to 98% and the absolute humidity ranged from 0.013 to 0.015 kg/m^3^.

**TABLE 1:** DNA-SIP experimental setup^*a*^

Expt	Culture	Reactor	C isotope	Day 0/day 5 temp (°C)	Total DNA mass collected (ng)	Day 5 estimated no. of methanotrophic cells^*b*^	Day 5 relative humidity (%)	Day 5 total no. of particles/L	Day 5 absolute humidity (kg/m^3^)
1	Maple leaf	1	12	24/NA^*c*^	16.2	9.05 × 10^0^	NA	NA	NA
		2	13	24/NA	18.3	ND^*d*^	NA	NA	NA
2	Maple	1	13	24/24	26	6.40 × 10^2^	81	3.30 × 10^5^	0.018
		2	12	24/24	8	3.27 × 10^1^	78	3.73 × 10^5^	0.017
3	Air	1	13	24/19	ND	NA	93	NA	0.015
		2	12	24/19	ND	NA	94	NA	0.015
4	Air	1	13	22/20	3	7.70 × 10^3^	86	3.99 × 10^5^	0.015
		2	12	22/21	11.8	1.45 × 10^5^	84	4.29 × 10^5^	0.015
5	Maple leaf	1	12	22/20	14.9	5.51 × 10^4^	80	2.58 × 10^5^	0.014
		2	13	22/19	28.6	3.01 × 10^5^	78	2.79 × 10^5^	0.013
6	Maple leaf	1	13	20/NA	23.4	NA	NA	NA	NA
		2	12	20/NA	19.2	NA	NA	NA	NA
7	Air	1	12	21/28	34.2	6.14 × 10^2^	86	3.31 × 10^5^	0.013
		2	13	20/18	7.4	1.70 × 10^2^	85	3.40 × 10^5^	0.013

a Details include airborne particle concentrations after 5 days, DNA mass extracted, estimated methanotrophic cells, and humidity present for seven experiments with airborne methanotrophs in duplicate bioreactors amended with either [^12^C]methane or [^13^C]methane.

b Based on number of *pmoA* genes detected by qPCR, assuming two copies of *pmoA* in a single methanotrophic cell (74).

c NA, not available. For experiments 1 and 6, the total number of particles and relative/absolute humidity were unknown; for experiment 6, the DNA extract was compromised.

d ND, not detected.

**FIG 4:**
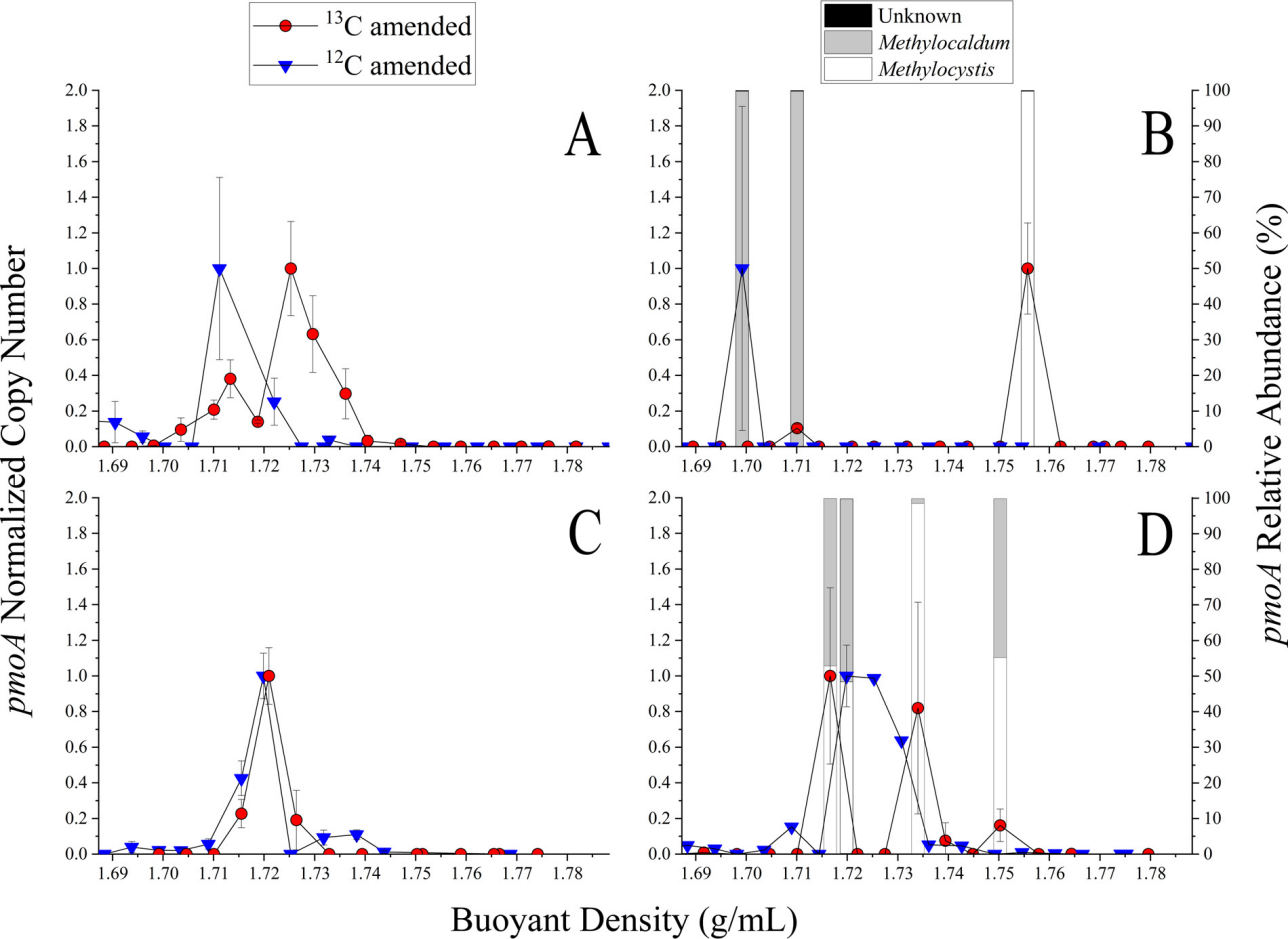
Isotopic incorporation of ^13^C into DNA from [^13^C]methane by aerosolized methanotrophic enrichment cultures. Each graph shows the average amounts of *pmoA* copies determined from technical replicates of a fraction with a specific buoyant density. The *pmoA* copy number was normalized to the highest copy number of the collected fractions for a sample (left *y* axis). Amplicons from qPCR of experiments 2 and 4 were sequenced (right *y* axis), and methanotrophs were identified. (A) Experiment 5; (B) experiment 2; (C) experiment 7; (D) experiment 4.

Duplicate bioreactor incubations with [^12^C]- or [^13^C]methane amendment were carried out through the entire DNA-SIP process for experiments 1, 2, 4, 5, and 7. Note that experiments 3 and 6 were not further processed (Table 1). Sequencing of *pmoA* gene amplicons was performed for selected fractions. DNA was present and *pmoA* genes were amplified in fractions with buoyant densities of 1.71 to 1.76 g/mL, with variable normalized *pmoA* gene copy numbers detected (Fig. 4).

Detection of growth via DNA-SIP analysis is dictated by detection of DNA at a higher density in a treatment receiving a ^13^C-labeled growth substrate than of the DNA of a treatment receiving a ^12^C-labeled growth substrate. Experiments 2, 4, and 5 exhibited a shift in the buoyant density of the DNA in this manner (Fig. 4A, B, and D). More specifically, the density of the ^13^C-treated samples of experiments 2 and 4 (Fig. 4B and D) have DNA of a similar density as the ^13^C-treated liquid-phase incubations at about 1.75 g/mL (Fig. 3). We therefore selected some fractions that corresponded with the liquid-phase results and sequenced the *pmoA* amplicons generated by qPCR. In experiments 2 and 4, *pmoA* gene ASVs associated with the genera *Methylocaldum* and *Methylocystis* were present at higher buoyant density fractions. These ASVs were also present in the less-dense DNA fractions in experiment 4 (Fig. 4D). The predicted PmoA sequences retrieved from heavier fractions also clustered with *Methylocystis* and *Methylocaldum* in a phylogenetic tree (Fig. S4). Overall, these results indicate the growth of methanotrophs on methane in the airborne state.

## DISCUSSION

### Methanotrophic diversity.

Based upon sequenced portions of the 16S rRNA gene and the *pmoA* gene, the maple leaf and air enrichments contained methanotrophs belonging to the family *Methylocystaceae* (type II). Additionally, the air enrichment contained members of the family *Methylococcaceae* (type I). The most dominant ASV of the 16S rRNA sequences of the *Methylocystaceae* were associated with *Methylocystis*. Specifically, this genus has been found in upland soils, rice paddies, groundwater, and other environments (41). The dominant ASV of the air enrichment culture was associated with *Methylocaldum* (type I) and specifically with a phylotype detected in a methanotrophic enrichment culture sampled from air over a landfill, which was able to oxidize methane under cloud-like conditions (39). In general, some members of the *Methylocaldum* genus are thermotolerant and have been found in various habitats, including landfills, upland soils, and rice paddies (41). It has been shown that the methanotrophs can be tolerant of desiccation during drought-like conditions and exposure to heat (42). Additionally, some methanotrophs form resting states and cysts (43), which could assist with their survival in the atmosphere and aeolian dispersal (39). Type II methanotrophs have been reported to tolerate dynamic and fluctuating desiccative conditions (42, 43), which are common in the phyllosphere and atmosphere. Type I methanotrophs are classified as being able to respond quickly when conditions become favorable and are classified as competitors/competitors-ruderals (44). Ecologically, there are benefits from both sets of attributes in terms of maintaining activity and growth in the atmosphere. Tolerating stress is essential to atmospheric life, but being able to respond quickly when a specific parameter, such as humidity or substrate concentration, becomes more conducive to growth might be beneficial too.

The substrate concentration needs to be adequate for metabolic activity. High- and low-affinity methanotrophs can metabolize and grow on low or high concentrations of methane, respectively. Some methanotrophs, including *Methylocystis* sp. strain SC2, have two isozymes of particulate methane monooxygenase that are expressed when exposed to different methane concentrations (45). Some “conventional” methanotrophs that were thought not to be able to oxidize atmospheric levels of methane can do so under certain conditions (37), but they are not known to grow on atmospheric methane. Currently, the only known isolate to grow on atmospheric levels of methane is *Methylocapsa gorgona* MG08, a type II methanotroph. This isolate is able to oxidize methane at high (e.g., 20% methane) and low atmospheric concentrations, with only a single particulate methane monooxygenase (36). There have been no demonstrated isolates of type I methanotrophs to grow on atmospheric methane. However, USCγ phylotypes were dominant in a cave that functions as a methane sink (46). A nearly complete genome of a *Methylocaldum*-like bacterium was found in soil in which atmospheric methane was reportedly oxidized (47). In our study, the “experiment 4 ^13^C 1” PmoA sequence had homology to that found in Methylocaldum gracile (see Fig. S4 in the supplemental material). The PmoA sequences from the airborne SIP experiments were homologous to those from experiments 2 and 4 as well as those from liquids 1 and 2.

Of the seven experiments in this study, only three results demonstrate synthesis of ^13^C-DNA and, presumably, growth of airborne methanotrophs on [^13^C]methane. Even though the concentration of methane did not reflect atmospheric concentrations, some methanotrophs (36) may be able to oxidize and grow on higher concentrations of methane, like those in our gas-phase incubation, as well as on lower atmospheric concentrations. Although our airborne incubations were performed at 1,500 ppm methane, almost 1,000 times higher than atmospheric concentrations, the data are still informative. Further work is needed to compare the methane oxidation abilities of type I and II methanotrophs as well as those of high- and low-affinity methanotrophs in the atmosphere at relevant concentrations.

### Physical retention of particles.

We aerosolized a mixture of a methanotrophic enrichment culture with Sphingomonas aerolata across multiple experiments into gas-phase bioreactors. As time went on in the process of introducing bioaerosols into the bioreactors, a dynamic equilibrium was reached, indicating that there was a limit to the number of particles that could be introduced into the bioreactors (Fig. 1A). After the introduction of particles, we found that bacterium-sized particles had a half-life of 3 days (Fig. 1B). The physical half-life of particles in rotating drums depends upon various factors, including rotation speed, humidity, and rotating drum design (48). The concentration of different-sized particles in rotating drums decreases over long periods of time through size changes via desiccation, humidification, agglomeration, or disintegration or through settling and impact with the sides of the bioreactor vessel.

### Factors affecting culturability.

Over the 5-day period, there was a substantial decrease in the concentration of particles across all size bins (Fig. 2C). However, we were able to cultivate bacteria from bioaerosols recovered from the bioreactors on DSMZ 921 agar when incubated in an anaerobic jar with 30% methane in the air of the jar over a 2-week period, after the 5-day aerial incubation. There was a significant difference in the numbers of culturable bacteria/particle initially and after the 5-day incubation period (Fig. S3). Staphylococcus epidermidis remained culturable after being suspended in a rotating drum for 5 days at 76% humidity (49). Sphingomonas aerolata remained culturable after 2 days at relative humidities between 81% and 97% in a previous study (18) that used the same gas-phase bioreactors described here. The present study demonstrated the survival of bacteria in air when the relative humidity was adjusted daily to be at least 95% (Fig. S3).

In our system, a variety of factors could affect culturability. The methods employed in experimental aerobiology to generate bioaerosols can negatively affect the integrity of the bacterial cell and, thus, culturability and growth potential. To decrease this, we utilized a single-pass aerosol generator (SPAG), which relative to conventional aerosolization methods was found to be the least damaging to bacteria (50). There was a decrease in the culturability of airborne bacteria based upon the ratio of culturable bacteria to particles in the 0.5- to 0.7-μm size bin. However, there are also salts present in the size bin, making a 1:1 ratio impossible (Fig. S2). Although washing the cell suspensions prior to aerosolization removes the salts, thus giving a more accurate bacteria-to-particle ratio, this imposes osmotic stress upon the bacteria. This imposed stress would negatively affect the growth potential of airborne cells. Benchmarking the number of culturable cells to the total number of cells would give insight about the culturable, the viable but nonculturable, and total populations.

In the environment, while microbes/bioaerosol particles are suspended in the air, they are subjected to numerous processes and stressors that age them. This aging process is driven by a variety of physical and chemical processes, all of which can affect the particle size and viability of the microbes (51, 52). For instance, the viability of Escherichia coli in a rotating drum was affected by solar radiation, humidity, ozone, and VOCs, which are part of the aging process in the alfresco atmosphere (53). In the experiments described here, bioaerosols were incubated in the dark, so humidity may be the primary environmental factor affecting the airborne cells in our study. Previous work has demonstrated that humidity has a large effect on bacterial culturability (54–56). The ability to respond to changing osmotic environments is important for the survival of airborne bacteria (57). During the incubation time in the experiments described here, humidity will affect the size of the particle and the amount of water associated with the bioaerosol particle (58), which could have implications for bacterial growth.

### Growth of methanotrophs in air.

The ability of a bacterium to grow in air depends on favorable conditions for growth and whether its residence time in air is long enough for the process of growth to occur. During the gas-phase incubations in this study, bacterium-sized particles had a half-life of about 3 days due to gravitational settling and impact with the sides of the bioreactor vessel. Mesophilic bacteria related to *Methylocystis* and *Methylocaldum* have growth rates between 0.068 and 0.074 h^−1^ at 30°C (45) and between 0.049 and 0.057 h^−1^ at 25°C (59), respectively, under optimal conditions in the liquid phase. Based upon this taxonomic association, the methanotrophic bacteria in the enrichment cultures utilized in this study had enough time to grow over the 5-day period, if conditions were favorable.

We conducted DNA stable-isotope experiments across seven experiments with two different methanotrophic enrichment cultures mixed with *S. aerolata*. Results from three experiments indicated that the airborne methanotrophs grew on [^13^C]methane (experiments 5, 2, and 4 as shown in Fig. 4A, B, and D, respectively). The denser DNA in experiments 2 and 4 contained ASVs associated with the genera *Methylocaldum* and *Methylocystis* (Fig. 4B and D). This suggests that *Methylocaldum* and *Methylocystis* grew on methane in the airborne state. Within the competitor-stress-ruderal (C-S-R) framework for methanotrophs, these genera were present separately across the spectrum of classification (44). The C-S-R framework puts methanotrophs into these categories related to survival and stress tolerance based upon known characteristics of the genera. According to this framework, the genera had the ability to tolerate stressors that were associated with biomass restriction (e.g., nutrient availability/fluctuation) and destruction (e.g., desiccation), which are important for airborne growth. For example, *Methylocystis* sp. SC2 tolerates changes in salinity (60). The constant evaporation and condensation of water on bioaerosol particles lead to osmotic shocks, so tolerating these conditions is important for atmospheric growth. In our experiments, the presence of these genera in the denser DNA similar in buoyant density to that of the liquid-phase incubations demonstrates that two rounds of growth likely occurred. The ability of these bacteria to tolerate stressors may have been key to their aerial growth.

The other experiment indicating incorporation of [^13^C]methane in air was experiment 5, in which there was a shift in the density of DNA between the ^12^C- and ^13^C-amended incubations. However, this shift in density was not as large as that observed in experiments 2 and 4. Based upon these data, it is possible that one round of growth or partial incorporation occurred for experiment 5. Depending upon the biochemical needs of the cell-facing airborne stressors, the fluxes of ATP and reducing equivalents could be diverted to processes other than growth. For growth to occur, new nucleotides need to be synthesized by the *de novo* pathway, which is more energy intensive, or the salvage pathway, which is less energy intensive (61). Since airborne cells are energetically limited, the salvage pathways might be favored over the *de novo* pathway, especially if DNA is damaged. It was found that excision repair might help bacteria to maintain viability after being in the air (62). However, excision repair occurs only across small portions of DNA (<50 bp), so it is unlikely that this would result in the shift in DNA density observed in this study. Traditional DNA-SIP relies upon at least two replications of cells for both strands of DNA to be replaced with ^13^C atoms. A single replication event of a cell would result in a ^12^C-^13^C hybrid double-stranded DNA. If DNA replication occurred only once in air, then partial incorporation could occur, which has been discussed in previous studies (63, 64). Further work is needed to confirm if the DNA molecules are composed of mixed carbon isotopes.

### Humidity and growth in air.

The variability in these results and the other gas-phase incubations could be due to many factors. When selected parameters of the gas-phase incubations were compared for all experiments, the absolute humidity values were the highest for experiments 2 and 4 (Table 1). Although experiment 5 had lower humidity and particle concentrations, the incorporation of [^13^C]methane could have occurred earlier in incubation, when the humidity was higher. Experiments 2 and 4 resulted in a higher absolute humidity at the end of the incubation period, which could have led to the results observed. As discussed previously, relative humidity is typically reported in studies focusing on bioaerosol survival, as it affects the water content of a bioaerosol particle (58). Since the temperature varied, the absolute humidity metric allows comparison of the experiments to one another.

The availability of water has been suggested to be a major regulator of microbial growth and activity (65). The minimum water activity for extremophilic microbial growth is about 0.6 (66), which corresponds to 60% relative humidity (17). Most bacteria can grow only at water activity levels greater than 0.91, but this varies depending on the specific bacterial strain and environmental conditions (67). Most importantly, it has been observed that there is a correlation between airborne microbial activity and humidity (17). Having a lower humidity also results in increased osmotic pressure (55), which could negatively affect growth potential. Based upon our data, a certain amount of water must be present to allow aerial bacterial growth. In this study, the particle size distributions demonstrate that the bacteria were not in large water droplets. However, the cells likely had at least a film of water surrounding part of the cells, which would be affected by the changing humidity levels in the bioreactors. This water on the cell surface would be an important interface allowing for partitioning of compounds that the cell could metabolize. Bacterial growth in water droplets under laboratory conditions (8) and microbial activity in cloud droplets (3) have been demonstrated. To our knowledge, this is the first study to demonstrate that bacteria grow in the air outside of water droplets under laboratory conditions.

### Conclusions, limitations, and future work.

This study examined the characteristics of aerosolized methanotrophic enrichment cultures and the potential growth of methanotrophic bacteria in the air. The ability to maintain culturability while in the airborne state has important consequences for aeolian dispersal of bacteria and also potential effects upon human health (49). It is also an important indication that a cell could have the potential to grow in air, depending upon environmental conditions. Only three out of seven experiments demonstrated methanotrophic growth in the airborne state as evidenced by production of heavier DNA. In our study, due to the challenges of aerosolizing and keeping the bacteria suspended in air, our samples contained limited biomass. The humidity decreased during the DNA-SIP incubations, which may have also affected bacterial growth. Although not all replicates support the hypothesis, the variation among the replicates might be explained through humidity.

Regardless, we demonstrate a proof of concept of the replication of aerial methanotrophic bacteria outside of water droplets. Future laboratory work should utilize higher humidity values and more sensitive techniques, such as the incorporation of radioactive elements, to detect airborne bacterial growth and activity. Additionally, atmospherically relevant methane concentrations should be used. This study used high methane concentrations, which may have affected which methanotrophs were cultured and identified. It is possible for a methanotroph to grow on high and low methane concentrations, so more work is needed to understand the growth of airborne methanotrophs. If they can metabolize and grow on methane, a very potent greenhouse gas, *in situ* in the atmosphere, then this would be a potential new methane sink. A better understanding of the spatial distribution of airborne methanotrophs and cellular activities of methanotrophs *in situ* while in the airborne state must be gained to truly evaluate the potential magnitude of this biological methane sink. In the ambient environment, these bacteria would also need sources of micro- and macronutrients for growth. In this study, the growth medium provided the necessary nutrients. The importance of different nutrient sources influencing aerial growth, including gaseous compounds, intracellular stores, and particles associated with cells, needs to be investigated. The activity of airborne microbes is understudied, and a combination of field sampling and experimental aerobiological techniques is necessary to answer questions central to the discipline of atmospheric microbiology.

## MATERIALS AND METHODS

### Establishment and incubation of methanotrophic enrichments.

An environmental enrichment culture was developed from a maple leaf collected from the Rutgers University, New Brunswick, NJ, campus. The leaf was rinsed with sterile water, and the rinsate was cultured with 10% (vol/vol, gas headspace) methane in DSMZ 921 medium. An ambient air enrichment culture was developed by sampling air above grass outside of a teaching and research building at Rutgers University, New Brunswick, NJ. The air was sampled into DSMZ 921 medium with a BioSampler (SKC, Inc., Eighty Four, PA), operating at 12.5 L/min, for a 2-h period. The sampling liquid was transferred into a 500-mL serum bottle and incubated statically at room temperature (~22°C) for 6 months with 10% methane. Later, prior to refreshing the culture, 2 mL of both cultures was transferred to triplicate 120-mL serum bottles with 40 mL DSMZ 921 broth. These cultures were amended with 4% methane (vol/vol, gas headspace) and incubated at room temperature with shaking at 100 rpm for 1 week. The methane in the headspace was measured with a gas chromatograph (described below), and the optical density of the culture at 600 nm was measured with an Evolution 60S UV-visible spectrophotometer (Thermo Scientific, USA).

Prior to the aerosol experiments described below, the initial enrichments were increased to a volume of 3 L by adding fresh, sterile DSMZ 921 medium, amended with 4% (vol/vol, gas headspace) methane, and transferred (10%, vol/vol) into 3 L fresh medium when needed for subsequent experiments after 2 weeks of incubation. Additionally, Sphingomonas aerolata NW12 (68) was cultured in a minimal medium (69) with 2 mM acetic acid at room temperature for 5 days prior to the start of each experiment. This organism was used as a negative control for aerosolized incubations because it does not metabolize methane.

### Analysis of methane.

Methane utilization by cultures in liquid was monitored with an Agilent 6890 gas chromatograph equipped with a flame ionization detector (GC-FID) with a GS-GasPro column (30 m long by 0.32 mm inside diameter; J&W Scientific, Folsom, CA), using previously described methods (70). Two alterations were made to this protocol: headspace samples (0.25 mL) were removed from culture vessels that were incubated at room temperature (~22°C), and the column was held isothermally at 210°C for 1.2 min. Standards were made in 125-mL serum bottles with 0.5, 1, 2, 3, 4, 6, 8, and 10% (vol/vol, gas headspace) methane.

### Bioaerosol measurement in experimental setup.

The particle size distributions of aerosols produced from the mixed cell suspensions of the methanotrophic enrichments and *S. aerolata* were assessed in an experimental setup previously described (71). Two liters of methanotrophic enrichment and 1 L of *S. aerolata* were centrifuged at 8,000 × *g* for 10 min and concentrated into 10 mL. Then, the cell suspension was split into two 5-mL samples. One sample was kept in medium, while the other sample was centrifuged (as described above), resuspended in sterile water, and centrifuged again. This was repeated three times, with a final resuspension in 5 mL of sterile water. Each of these was aerosolized into the experimental setup with the single-pass aerosol generator (SPAG) (50) by injecting the cell suspensions at a rate of 0.1 mL/min with a syringe pump into the SPAG. The bioaerosols were generated at a flow rate of 1.2 L/min of HEPA-filtered air and mixed with humidified HEPA-filtered air flowing at 20 L/min. The system was maintained at 18 to 20 lb/in^2^. The generated bioaerosols were directed into the experimental setup. The washed and unwashed samples were aerosolized for 30 min each, and the particle size distributions were measured once in 5-min increments (0 to 5 min, 5 to 10 min, etc.) with an ARTI HHPC-6 hand-held airborne particle counter (Met One, Grants Pass, OR). Washing the cell suspension was done to remove the salts from the cultivation medium, since those particles would be detected as well.

### Filling gas-phase bioreactors, culturability, and biomass testing.

The retention, culturability, and particle size distributions of the methanotrophic air enrichment mixed with a culture of *S. aerolata* were assessed. Two liters of the methanotrophic air enrichment culture and 1 L of *S. aerolata* were combined and concentrated by centrifugation into ~8 mL. This cell suspension was aerosolized using the SPAG, and the bioaerosols were directed into duplicate 320-L gas-phase stainless steel bioreactors (1.02 m long by 0.66 m in diameter) (18) over a 60-min period, with each bioreactor receiving bioaerosols for 5 min alternately during that time. The reactors were rotating at 1 rpm for the time of filling, and rotation was increased to 3 rpm afterwards for the duration of incubation. While each reactor was receiving bioaerosols, the particle size distribution was measured at the bioreactor outlet with an ARTI HHPC-6 (Met One, Grants Pass, OR) to assess a filling profile every 5 min. After filling of the gas-phase bioreactors, the particle size distributions were measured daily for 5 days. The relative humidity was measured using the ARTI HHPC-6 and adjusted to 95% as needed by using previously described methods (18). Five days was selected as the incubation time, because the average residence time of bacteria in the atmosphere is 5.6 days (26). Due to temperature variations during incubation at room temperature, the relative humidity readings were converted to absolute humidity using an online calculator (https://planetcalc.com/2167/) to allow for comparison. The experiments described here were conducted twice for duplicate bioreactors (*n* = 4). Prior to each incubation, the bioreactors were disinfected with 70% (vol/vol) ethanol and flushed with HEPA-filtered air for 3 h at a flow rate of ~30 L/min.

For one experiment, after introduction of the bioaerosols into the gas-phase bioreactors, BioSamplers containing 5 mL DSMZ 921 medium were used to sample the bioreactors for 1 min. This was repeated after the 5-day incubation period. This sampling liquid was used to culture aerosolized methanotrophs on DSMZ 921 agar plates in an anaerobic jar filled with 30% methane. The numbers of CFU on the agar plates were counted after 2 weeks of incubation at room temperature and converted into CFU/L by dividing by the total amount of air sampled, 12.5 L. The CFU/L metric was normalized by the number of particles/L, detected with the optical particle counter in the 0.5- to 0.7-μm size bin, to present the data as CFU/particle.

### Gas-phase stable-isotope probing.

The methanotrophic enrichments and *S. aerolata* cultures were incubated as described above. The bioreactors were filled with bioaerosols generated from a final 8-mL cell suspension derived from 2-L methanotrophic and 1-L *S. aerolata* cultures. The operating and filling procedures were the same as described above. One bioreactor was amended with 1,500 ppmv [^12^C]methane (AirGas, Radnor, PA), and the other was amended with 1,500 ppmv [^13^C]methane (Sigma-Aldrich). The bioaerosols were incubated in the bioreactors for 5 days, with no humidity adjustments during incubation, after which the aerosols were sampled onto membrane filters (0.8-μm SUPOR filters; Pall Life Sciences, Port Washington, NY) at a flow rate of 20 L/min for 1 h and stored at −80°C until DNA extraction.

### Liquid-phase stable-isotope probing.

In addition to gas-phase incubations, we conducted liquid-phase DNA-SIP as a control. Two duplicate 10% (vol/vol) transfers of the methanotrophic air enrichment culture were established for a final volume of 40 mL in 120-mL serum bottles. One duplicate set was amended with 4% (vol/vol, gas headspace) [^12^C]methane, and the other set was amended with 4% (vol/vol, gas headspace) [^13^C]methane in DSMZ 921 medium. The concentration of the methane in the duplicate bottles was measured on days 0, 1, 5, 6, and 7, as described above. After 7 days, the cells were centrifuged at 8,000 × *g* for 10 min, and the resulting cell pellets were stored at −20°C until DNA extraction.

### DNA extraction.

DNA was extracted by following a phenol-chloroform-based extraction method for bioaerosols on filters with slight modifications (18). Briefly, filters were wetted with 50 μL of a solution of glucose (50 mM), EDTA (10 mM), and Tris-HCl (25 mM) and then subjected to 5 cycles of freeze-thawing. Freezing was done by submerging the tube containing the filter in −80°C-chilled 100% ethanol, and thawing was done by placing the tube in a 55°C heat block. Following this, 300 μL of 0.4 mg/mL lysozyme and 50 μL of 0.5 M EDTA were added and incubated at room temperature for 5 min at 100 rpm. Then, 50 μL of 10% SDS and 800 μL of phenol-chloroform-isoamyl alcohol (25:24:1) were added. The tube was vortexed to form an emulsion and then centrifuged at 16,000 × *g* for 3 min. The aqueous phase of the emulsion was transferred to a new tube, extracted with 800 μL of phenol-chloroform-isoamyl alcohol (25:24:1), and centrifuged again. The DNA from the final aqueous layer was precipitated with 30 μL of 3 M sodium acetate, 1 mL of 100% ethanol, and 5 μL of glycogen (5 mg/mL). This tube was centrifuged at 16,000 × *g* for 15 min at 4°C. The pellet was resuspended in diethyl pyrocarbonate (DEPC)-treated sterile water. The liquid-phase stable-isotope probing samples of the air enrichment were extracted with a DNeasy UltraClean microbial kit (Qiagen, USA) in accordance with the manufacturer’s protocol, with the DNA suspended in 30 μL of nuclease-free water. All DNA samples (1 μL) were quantified with a Qubit double-stranded DNA high-sensitivity assay kit and a Qubit 2.0 fluorometer (Invitrogen, OR, USA).

### Gradient formation and fractionation.

^12^C- and ^13^C-labeled DNA were separated on a CsCl gradient. The CsCl solution was prepared in 0.1 M Tris-EDTA (TE) buffer, resulting in a buoyant density of about 1.7742 g/mL as measured by an AR200 digital refractometer (Reichert Technologies, NY), and was distributed into 4.7-mL OptiSeal polypropylene ultracentrifuge tubes (Beckman Coulter, CA). All the genomic DNA (gDNA) from the gas-phase incubations and 1 μg of the gDNA of the liquid-phase incubations of the air enrichment were added to the CsCl gradients. The buoyant densities of the gradients were then adjusted to be between 1.7231 and 1.7340 g/mL by the addition of more of the CsCl solution or TE buffer. The gradients were centrifuged at 86,700 × *g* at 28°C for at least 48 h in an Optima Max TL ultracentrifuge (Beckman Coulter, CA). After centrifugation, the samples were fractionated with a fraction recovery system (Beckman Coulter, CA), with the aid of a syringe pump, and 24 fractions of 200 μL were collected. The DNA was precipitated using a glycogen-assisted ethanol precipitation. After precipitation, DNA was resuspended in 20 μL of nuclease-free water.

### qPCR of fractions.

From the gDNA of the enrichment cultures, a portion of the *pmoA* gene was amplified with primers A189F/mb661R (72–74) in accordance with previous protocols (75). The amplicon was ligated into the pCR2.1 TOPO vector (Invitrogen, Carlsbad, CA) and transformed into TOP10 E. coli made competent by following the protocols of Inoue et al. (76). Plasmids were extracted with a Zyppy plasmid miniprep kit (Zymo Research, Irvine, CA) from transformed cells incubated overnight in LB broth supplemented with 50 μg/mL kanamycin. The presence of the inserted amplicon in the plasmid was verified by PCR and visualized on a 1.5% (wt/vol) agarose gel. Plasmids were linearized with FastDigest KpnI (Thermofisher Scientific, MA) in accordance with its associated protocol. The linearized plasmid was purified with a QIAquick PCR purification kit (Qiagen, USA) and quantified with a Qubit 2.0 fluorometer with dsDNA broad-range standards (Invitrogen, Carlsbad, CA) by following the manufacturer’s protocols. The abundance of the *pmoA* gene was assessed by qPCR in accordance with the protocols of Kolb et al. (75). The reactions were performed in 10 μL with 5 μL PowerUp SYBR green master mix (ThermoFisher Scientific), 0.67 μM each primer, 0.5 μL 25 mM MgCl_2_, 2.16 μL water, and 1 μL of template. The standard curves of the linearized plasmids were a series of 10-fold dilutions with an average amplification efficiency of 80.9% and an average *r*^2^ value of 0.98, with the detection limit ranging between about 10^2^ to 10^3^ total *pmoA* copies. The average copy numbers of *pmoA* in the collected fractions from the DNA-SIP experiments were quantified as the average of technical replicates with a standard curve associated with each qPCR plate. Experiment 2 was analyzed by qPCR with a total reaction volume of 50 μL with 1 μL of template, but the concentrations of the other components remained the same.

### Sequencing and bioinformatics.

DNA extracts from maple leaf and air enrichments were sent for PCR amplification and sequencing of the V4 region of the 16S rRNA gene by Mr. DNA (Shallowater, TX, USA) (https://www.mrdnalab.com). Using the primer set 515f/806r (77), a portion of the 16S rRNA gene was amplified with a HotStarTaq plus master mix Kit (Qiagen, USA). The following conditions were used for PCR amplification: 94°C for 3 min, followed by 30 cycles of 94°C for 30 s, 53°C for 40 s, and 72°C for 1 min, and ending with a final elongation at 72°C for 5 min. An Ion S5 XL instrument (Ion 530 chip) was used to sequence the amplicons in accordance with the manufacturer’s guidelines by Mr. DNA (Shallowater, TX, USA). Selected amplicons from the *pmoA* qPCR analysis of DNA-SIP fractions were purified with a QIAquick PCR purification kit (Qiagen, USA). The amplicons were confirmed on a 1.5% (wt/vol) agarose gel and sent to Mr. DNA for Illumina MiSeq 2 × 300 bp sequencing.

The 16S rRNA and *pmoA* gene sequences were demultiplexed with sabre (https://github.com/najoshi/sabre). All fastq files were imported into QIIME2 (v2020.2) (78). The 16S rRNA gene sequences were trimmed and denoised with DADA2 (79), and the *pmoA* sequences were trimmed and denoised in vsearch (80). A naive Bayes scikit-learn classifier (81, 82) for the 16S rRNA gene primer set was trained on the extracted reference full-length operon reads of the Silva v132 database (83) and used for classification of the ASVs. The gene sequences were grouped at the family level (for 16S rRNA gene) or the genus level (for the *pmoA* gene) for visualization. The relative abundances were calculated manually. ASVs that were of interest were manually queried against the NCBI (https://www.ncbi.nlm.nih.gov) BLAST database with the BLASTn algorithm. Samples were rarefied and analyzed for Shannon’s diversity, Chao1, and observed ASVs in QIIME2 (v2020.2) (see Table S1 in the supplemental material). The *pmoA* sequence-naive Bayes scikit-learn classifier was trained with the primers used for PCR on a *pmoA* database (84).

The most abundant *pmoA* ASV in the ^13^C incubations of liquid 1, liquid 2, experiment 2, and experiment 4 were selected. MEGA11 (85) was used to translate the DNA sequences into amino acids and aligned with Clustal W (86). Phylogenetic trees were made with a maximum-likelihood tree computed with the Jones Taylor Thornton matrix-based model of amino acid substitution with 1,000 bootstraps. PmoA sequences used for comparison were accessed from GenBank with the corresponding accession numbers shown in Fig. S4.

### Statistical analysis.

OriginPro 2020b was used to conduct a *t* test to compare the culturability of the methanotrophic air enrichment culture after aerosolization with that after 5 days of suspension in air.

### Data availability.

All sequences have been deposited in the NCBI Sequence Read Archive (SRA) under accession no. PRJNA863376.
