# Supplementary material for: Characterization and DNA Stable-Isotope Probing of Methanotrophic Bioaerosols
Source: Microbiol Spectr. 2022 Nov 21;10(6):e03421-22. doi: 10.1128/spectrum.03421-22 (PMC9769660; doi:10.1128/spectrum.03421-22)
Supplement: Supplemental file 1 — Supplemental material. Download spectrum.03421-22-s0001.pdf, PDF file, 0.4 MB [file spectrum.03421-22-s0001.pdf]

Characterization and DNA stable isotope probing of methanotrophic bioaerosols

Kevin P. Dillon, Valdis Krumins, Aishwarya Deshpande, Lee J. Kerkhof, Gediminas Mainelis, and Donna E. Fennell

SUPPLEMENTAL MATERIAL

Table S1: Diversity metrics of the *16S rRNA* gene reads of the methanotrophic enrichment cultures. All metrics were calculated in QIIME2 and rarefied at 3000 reads.

Enrichment Culture	Observed ASVs	Shannon's Diversity	Chao1
Air	41	3.3	51
Maple Leaf	23	2.5	23

Table S2: The particle concentration (particles/L; average ± standard deviation) of the gas-phase bioreactors after introducing bioaerosols during characterization experiments.

Size Bin (μm)	Optical Diameter (μm)	Particles/L					
		Day 0	Day 1	Day 2	Day 3	Day 4	Day 5
0.3 – 0.5	0.4	$4.4 \times 10^5 \pm 6.6 \times 10^3$	$3.9 \times 10^5 \pm 2.8 \times 10^4$	$3.5 \times 10^5 \pm 3.4 \times 10^4$	$3.2 \times 10^5 \pm 4.7 \times 10^4$	$2.8 \times 10^5 \pm 5.5 \times 10^4$	$2.3 \times 10^5 \pm 5.2 \times 10^4$
0.5 – 0.7	0.6	$3.4 \times 10^5 \pm 2.1 \times 10^4$	$2.7 \times 10^5 \pm 1.8 \times 10^4$	$2.1 \times 10^5 \pm 2.5 \times 10^4$	$1.8 \times 10^5 \pm 2.8 \times 10^4$	$1.5 \times 10^5 \pm 3.2 \times 10^4$	$1.0 \times 10^5 \pm 2.8 \times 10^4$
0.7 – 1	0.85	$2.1 \times 10^5 \pm 3.3 \times 10^4$	$1.5 \times 10^5 \pm 2.1 \times 10^4$	$1.0 \times 10^5 \pm 1.9 \times 10^4$	$8.2 \times 10^4 \pm 1.5 \times 10^4$	$6.3 \times 10^4 \pm 1.5 \times 10^4$	$3.9 \times 10^4 \pm 1.2 \times 10^4$
1 – 2	1.5	$1.2 \times 10^5 \pm 3.2 \times 10^4$	$8.1 \times 10^4 \pm 2.0 \times 10^4$	$4.9 \times 10^4 \pm 1.2 \times 10^4$	$3.6 \times 10^4 \pm 7.9 \times 10^3$	$2.6 \times 10^4 \pm 7.2 \times 10^3$	$1.5 \times 10^4 \pm 5.2 \times 10^3$
2 – 5	3.5	$3.1 \times 10^4 \pm 1.4 \times 10^4$	$1.7 \times 10^4 \pm 6.9 \times 10^3$	$7.7 \times 10^3 \pm 2.8 \times 10^3$	$5.3 \times 10^3 \pm 1.6 \times 10^3$	$3.5 \times 10^3 \pm 1.1 \times 10^3$	$1.8 \times 10^3 \pm 7.8 \times 10^2$
> 5	7.5	$8.6 \times 10^2 \pm 7.3 \times 10^2$	$2.5 \times 10^2 \pm 1.8 \times 10^2$	66 ± 43	38 ± 16	19 ± 14	7.8 ± 7.9

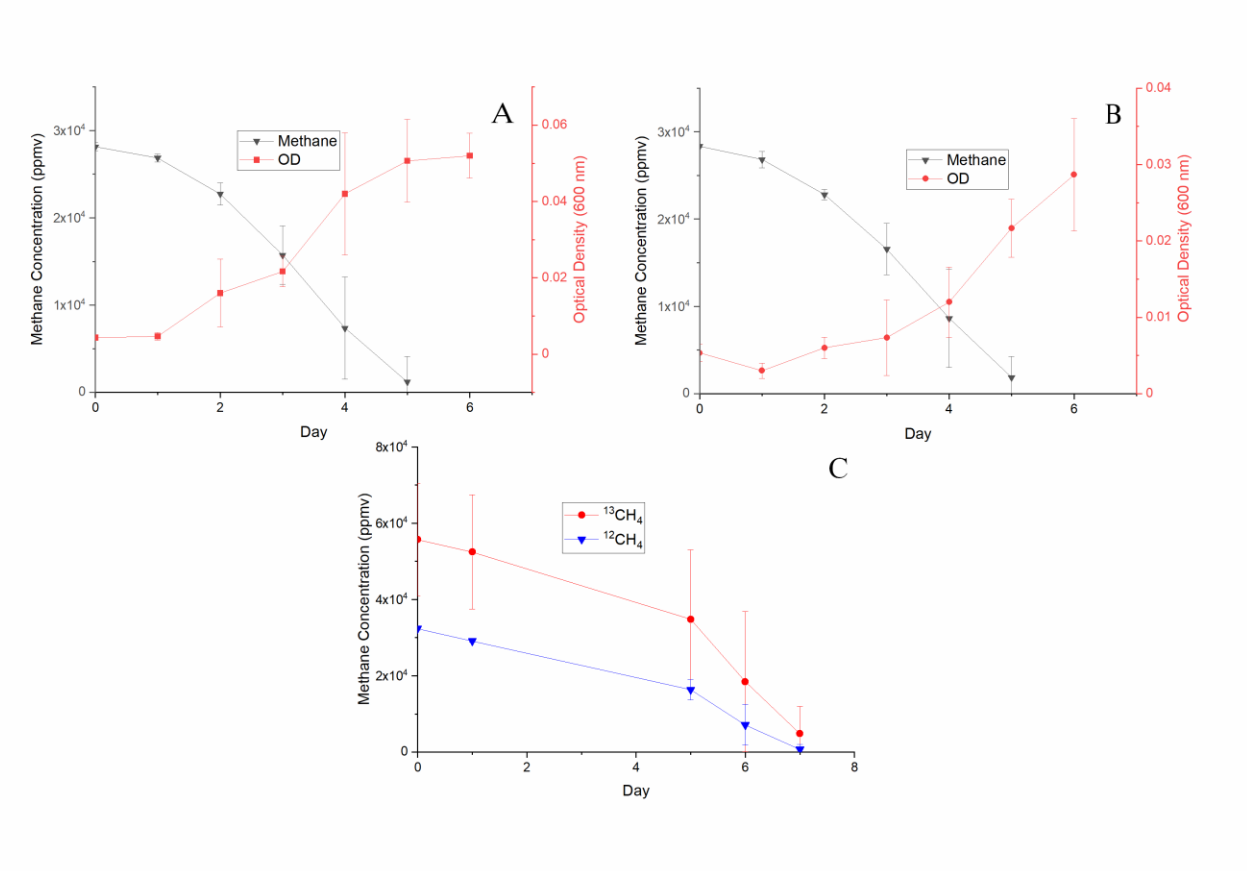

Figure S1: Methanotrophic enrichment culture cell growth. The growth of the air enrichment culture and loss of methane in triplicate incubations (A). The growth of the maple leaf enrichment culture and loss of methane in triplicate incubations (B). The loss of methane by the air enrichment culture for the liquid-phase DNA-SIP experiment (C).

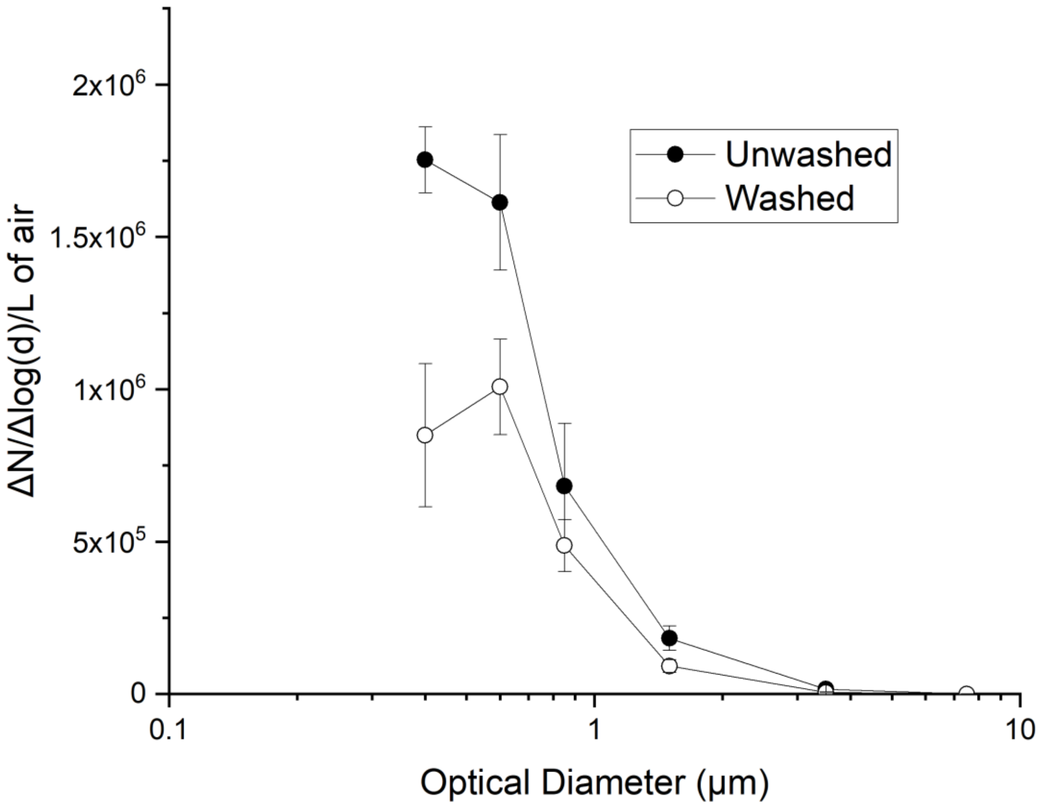

Figure S2: The particle size distribution of the methanotrophic air enrichment culture when washed with sterile water and when unwashed (still containing growth medium salts).

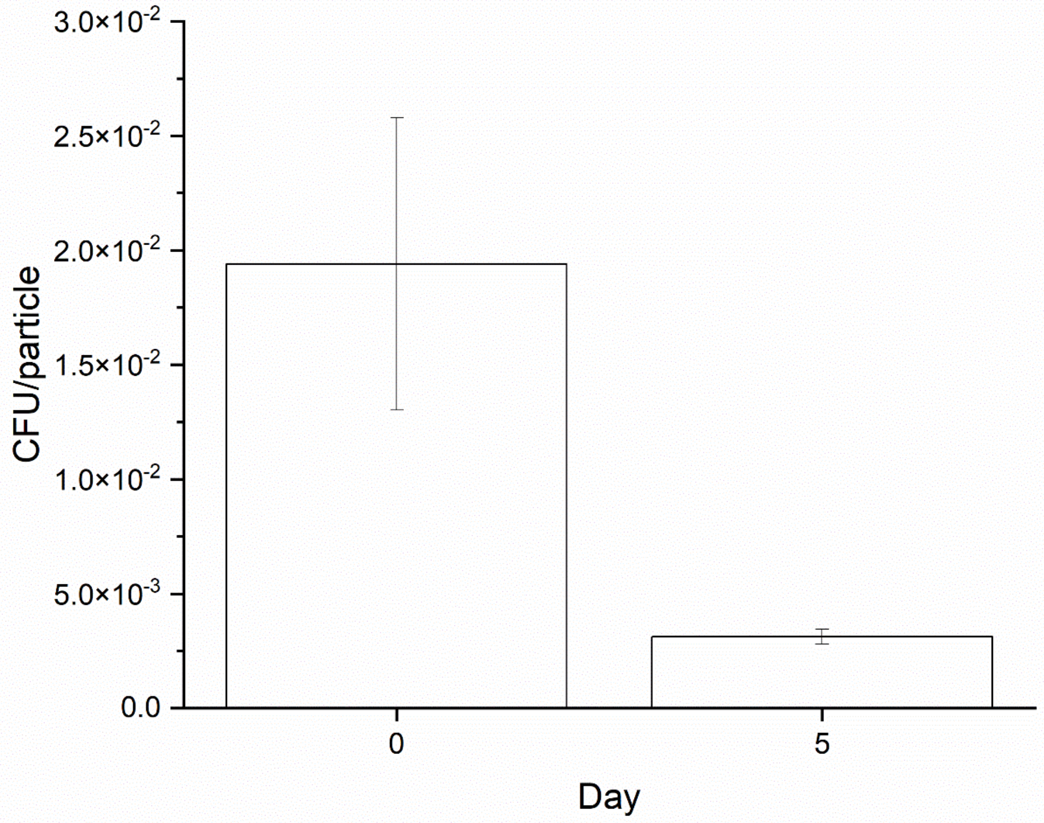

Figure S3: Average colony forming units (CFU) per the number of particles in the 0.5 – 0.7 µm size bin on Day 0 and after five days of incubation (Day 5). The bacteria were derived from the methanotrophic enrichment cultures and cultured on DSMZ 921 agar, but their identity as methanotrophic bacteria was not confirmed.

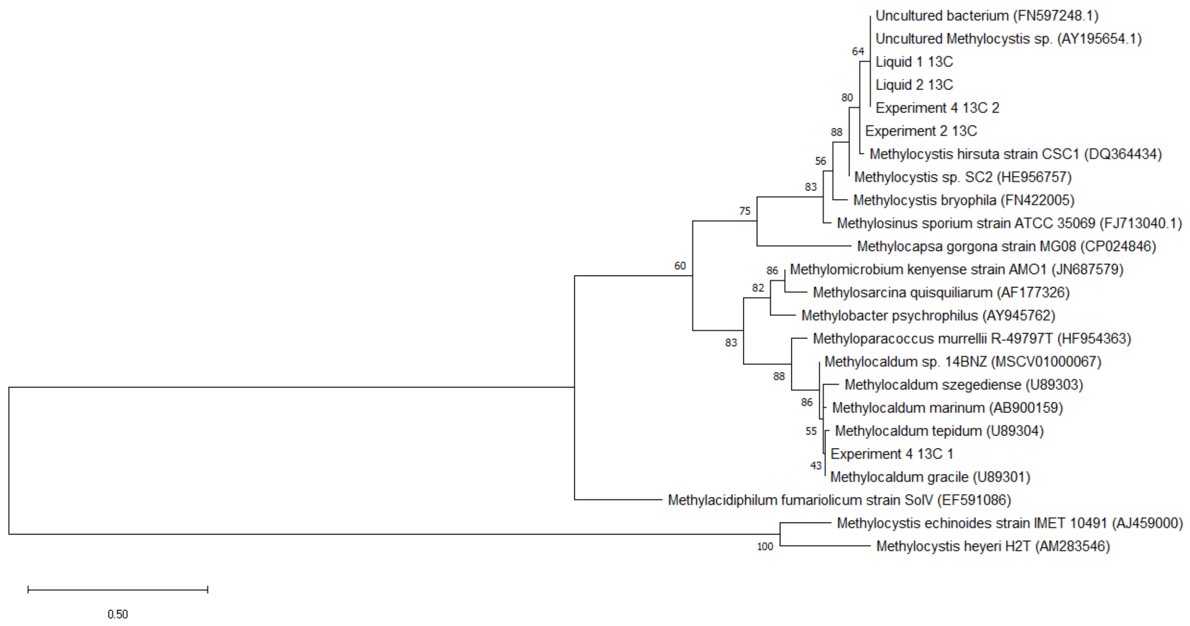

Figure S4: Phylogenetic tree based on predicted PmoA. The top *pmoA* gene ASV in each of the ^{13}C incubations were selected, translated to the corresponding amino acid sequence, and placed in a phylogenetic context with the MEGA11 software. Experiment 4 had two abundant ASVs designated as different genera and were designated as “1” and “2.” NCBI accession numbers for the comparative PmoA sequences are in parentheses.
